# Evaluation of Epigenetic and Radiomodifying Effects during Radiotherapy Treatments in Zebrafish

**DOI:** 10.3390/ijms22169053

**Published:** 2021-08-22

**Authors:** Gaia Pucci, Giusi Irma Forte, Vincenzo Cavalieri

**Affiliations:** 1 Department of Biological, Chemical and Pharmaceutical Sciences and Technologies (STeBiCeF), University of Palermo, 90128 Palermo, Italy; gaia.pucci91@gmail.com; 2 Institute of Molecular Bioimaging and Physiology, National Research Council, 90015 Cefalù, Italy; 3 Zebrafish Laboratory, Advanced Technologies Network (ATeN) Center, University of Palermo, 90128 Palermo, Italy

**Keywords:** epigenetics, radiomodifiers, radiotherapy, zebrafish, embryogenesis

## Abstract

Radiotherapy is still a long way from personalizing cancer treatment plans, and its effectiveness depends on the radiosensitivity of tumor cells. Indeed, therapies that are efficient and successful for some patients may be relatively ineffective for others. Based on this, radiobiological research is focusing on the ability of some reagents to make cancer cells more responsive to ionizing radiation, as well as to protect the surrounding healthy tissues from possible side effects. In this scenario, zebrafish emerged as an effective model system to test for radiation modifiers that can potentially be used for radiotherapeutic purposes in humans. The adoption of this experimental organism is fully justified and supported by the high similarity between fish and humans in both their genome sequences and the effects provoked in them by ionizing radiation. This review aims to provide the literature state of the art of zebrafish in vivo model for radiobiological studies, particularly focusing on the epigenetic and radiomodifying effects produced during fish embryos’ and larvae’s exposure to radiotherapy treatments.

## 1. Introduction

### 1.1. The Role of Radiotherapy in Cancer Treatment

In the most common radiation-therapy (RT) approach used in the clinical setting, an external beam delivers high-energy rays (photons, protons or particle radiation) to the tumor. Ionizing radiation (IR), used in conventional RT, consists of high- or low-energy photon/electromagnetic beams (X- and ɣ-rays), with typical lineareEnergy Ttansfer (LET) values in the range of 0.2 to 5 keV/µm. This allows the delivery of controlled doses of radiation to kill a defined tumor mass, limiting collateral damage to the surrounding tissue [1].

In the last 25 years, a strong technological advancement has been developed, allowing modern RT equipment to release more specific and targeted dose intensities, with concave or complex shapes [2]. Nevertheless, RT plans for distinct tumor types are still customized exclusively from a conformational point of view, not considering the clinical and molecular heterogeneity of the tumors [3]. Consequently, nonaggressive tumors receive an overdose, with side effects on the surrounding healthy tissues, while the more aggressive tumor subtypes receive an total dose insufficient to avoid the onset of loco-regional recurrence due to tumor radioresistance [4]. It follows that the personalization of therapeutic plans primarily requires improving the cancer cells’ radiosensitivity. Among the methods explored, one attractive possibility is given by the combination of RT and the use of molecules able to increase the RT success. Accordingly, in recent years the attention has been strongly focused on the use of either natural compounds or anthropogenic chemicals as RT coadjuvants, with the aim of increasing both normal tissue radioprotection and tumor cell radiosensitization [5,6]. While radioprotectors are often antioxidant compounds designed to reduce the damage in normal and tumor tissues caused by radiation, radiosensitizers increase the indirect radiation effects by promoting free-radical fixation or inhibiting activation of detoxification pathways.

### 1.2. Zebrafish as a Model in Radiobiology

Most of the radiobiological experimentation is carried out on different types of primary or immortalized human or animal cell cultures. To reach valid conclusions, however, this approach needs to be complemented by further analysis, using appropriate in vivo preclinical models that take into account the complex physiological phenomena related to organisms in their entirety. Among mammalian models, the mouse (*Mus musculus*) is widely used, due to several advantages, such as their high genetic similarity with humans, and the possibility of conducting cell type-specific knockout experiments and electrophysiology and behavioral studies [7]. In addition, mice are well-established models for reverse genetics, aging and injury paradigms, allowing access to a large collection of mutants and antibodies [8]. On the other hand, their major disadvantages relate to their high cost, the frequent maintenance and manual cleaning of the cages, their relatively long gestation time of about 20 days, and parental sacrifice, in the case of genetic manipulation or other experimental evaluations at the early stages of development.

The zebrafish (*Danio rerio*) model provides the opportunity to bridge the ever-present gap between in vitro studies and rodents. Zebrafish, a small fish living in Southeast Asian freshwater habitats, is increasingly used as an in vivo study model in the context of basic research, toxicology and translational medicine [9]. Being a vertebrate, it shows a close genetic relationship to humans, with a shared 70% zebrafish–human overall orthology assignment [10]. In this regard, the zebrafish is an excellent experimental tool for human cancer research, essentially, because many key genes involved in cell cycling, oncogenesis and tumor suppression are conserved between the two species [10,11]. Additional advantageous features make zebrafish a valid alternative to the conventional mouse model for the development of numerous therapeutic methods, including RT. First, these fish are quite small (adults are about 2–3 cm long), of ready abundance and accessibility and they can be easily maintained in the laboratory without excessive cost. Their high fertility rate, combined with short generation time and large number of offspring, reduces the time to produce experimental replicates, thereby increasing their potential for statistical validity. It follows that zebrafish embryos provide a rapid and simple system to screen novel agents to be used as radio-protectors/sensitizers.

Importantly, numerous approaches have been developed to reproduce and study human cancer diseases, for example, through the localized microinjection of tumor cells into zebrafish and subsequent pharmacological approaches aimed at evaluating possible therapeutic protocols by chemical screens and in vivo imaging [12]. Thanks to the external fertilization and optical clarity of wild-type embryos and some adult mutant lines, the in vivo non-invasive visualization of fluorescent, tagged cells, or the evaluation of processes, such as angiogenesis, tumor growth/reduction or cell-cell interaction, have become simplified assays [13]. Worth mentioning, the immature adaptive immune system of zebrafish embryos accepts the transplantation and survival of human cancer cells with no need for immunosuppression [14]. In addition, their main organs, such as the brain, eyes, spinal cord, vasculature and hematopoietic systems, are already defined at 48 h post-fertilization (hpf)—a much shorter time than that of mice—allowing rapid evaluation of RT efficacy on early embryos. The choice of the zebrafish embryo is ideal for this kind of study, since embryogenesis is the most radiosensitive stage of the vertebrate life cycle, due to rapid cell division [15]. Furthermore, the aqueous environment in which the embryos develop promotes homogeneous distribution of the irradiation dose. Finally, thanks to an irradiation size less than 1 mm between the cell monolayer culture and subcutaneous tumors or normal tissue organs, zebrafish embryos could be used to study relative biological effectiveness (RBE) and for detailed investigations on the RBE–LET relationship, both for low- and high-LET radiation types [16].

## 2. Epigenetic Changes Inflicted by Radiation during Zebrafish Embryogenesis

Irradiation of zebrafish embryos determines a series of specific morphological anomalies during development, including spinal curvature, body-length shortening, pericardial edema, inhibition of yolk-sac resorption, microphthalmia and microcephaly [11,17]. Additional effects pertain to variation in mortality and hatching rates, behavioral activity and the expression of genes related to DNA damage, cell death and detoxification pathways [18]. Altogether, these abnormalities are broadly comparable to those observed in mammals exposed to therapeutic or accidental radiation [19,20]. Numerous studies have also shown that fish cells not directly traversed by IR exhibit similar responses to those of directly irradiated cells, a phenomenon already known as the bystander effect [21]. This effect relies on cell communication through gap junctions or by the emission of soluble factors, such as reactive oxygen species (ROS) or cytokines, and could eventually lead to cell death, neoplastic transformation, and genomic instability [21].

Several studies have suggested that genomic instability induced by IR could be related to epigenetic effects, since IR are able to induce modulation of spatiotemporal gene expression patterns without variation in the DNA sequence. In this respect, it is widely recognized that mitochondria play a fundamental role in the IR response, and that dysfunction of these organelles leads to oxidative stress which, in turn, affects epigenetic regulation [22].

In zebrafish, epigenetic marks such as DNA methylation, histone post-translational modifications and microRNA relative abundance undergo normal changes during embryogenesis [13,23]. For example, the global 5-methylcytosine level starts to increase after 3.3 hpf and reaches a plateau between 6 and 96 hpf. Accordingly, knock down of DNA methyltransferases (DNMT) -1 and -3b, by means of antisense morpholino oligonucleotides, confirmed the importance of these two enzymes during zebrafish embryogenesis. In fact, DNMT1 appears to play the role of an essential genome regulator, because it modulates the DNA double-strand-breaks-repair rate and suppresses the abnormal activation of the DNA damage response (DDR) in the absence of exogenous injury [24].

Compared with unperturbed control embryos, exposure to ɣ rays at 0.7, 7, 70 and 550 mGy/d induced a statistically significant dose-dependent increase in DNA cytosine methylation, both in directly irradiated embryonic zebrafish fibroblasts or in cells subjected to bystander effects [25]. A concordant study showed the radiosensitizing effect of pretreatment, with the DNA methylating agent Temozolomide, before the administration of 10 Gy ɣ rays on 24 hpf zebrafish embryos containing U251 xenotransplants [26].

Covalent post-translational modifications (PTMs) of specific amino acid residues on histones are frequently associated with either normal or IR-induced changes between transcriptionally permissive and repressive chromatin states in a wide variety of cell types and organisms, including zebrafish [27,28]. A recent study showed that exposure of early zebrafish embryos to 32.7 mGy ɣ rays caused a specific enrichment of histone H3 trimethylation at lysine 4 (H3K4me3), 9 (H3K9me3) and 27 (H3K27me3) on three genes localized on distinct chromosomes, namely hepatocyte nuclear factor 4 alpha, geminin DNA replication inhibitor, and vascular endothelial growth factor Ab [29]. In particular, the increased amount of the negative epigenetic marks H3K9me3 and H3K27me3 suggests that IR could mainly impact on heterochromatinization as a mechanism for genome repression [29].

Finally, although microRNAs are absent in the early fish zygote, miR-125b acts as a negative regulator of the p53 network, both in zebrafish and in human cells [30]. In fact, p53 induction and apoptosis are typical outcomes following miR-125b knockdown [31]. Importantly, a significant downregulation of miR-125b and a concomitant increase in the p53 protein level have been detected in 24 hpf zebrafish embryos exposed to 40 Gy ɣ rays, confirming the importance of miR-125b in the modulation of apoptosis in zebrafish embryos exposed to IR [30].

In recent years, transgenerational consequences of epigenetic modifications in zebrafish, due to IR, have caught the attention of researchers. In this regard, Kamstra JH et al. [32] studied the relationship between IR-induced changes in DNA methylation and gene expression, and their persistence over fish generations. In this study, adult fish were exposed to a total dose of 5.2 Gy for 27 days and maintained for 1 year before generating F1, F2 and F3 by family inbreeding. Global epigenetic analysis revealed that the chromatin of F1 embryos, derived from irradiated founder fish, was highly enriched in H3K4me3, H3K27me3 and H3K4me1 and exhibited higher numbers of differentially methylated regions (DMRs), compared with control embryos. In particular, 62% of the DMRs located upstream to transcriptional start sites were associated with expressed genes, of which 49% were differentially expressed. Locus-specific analysis on selected genes involved in metabolic pathways, Wnt signaling, focal adhesion, calcium signaling and MAPK signaling, revealed that the above-mentioned changes in DNA methylation persisted in the chromatin of F2 and F3 embryos, highlighting the involvement of DNA methylation in transgenerational effects caused by IR. Martin L. et al. [33] analysed the gamma radiation effect on the non-coding transcriptome in the first-generation offspring of exposed parents. In particular, one year after parental exposure (6 months zebrafish, 8.7 mGy/h for 27 days; 5.2 Gy total dose), fish were mated and non-coding RNA expression profile was analysed in F1 embryos (5.5 hpf) by high-throughput sequencing. Using previous F1-γ genome-wide gene expression data, hundreds of mRNAs were predicted as targets of differentially expressed (DE) miRNAs. The DE analysis between F1offspring of gamma-exposed parents and those of controls showed 22 DE miRNAs, of which 55% were up-regulated and involved in pathways related to cancer processes, such as the control of pluripotency, proliferation and metastasis. Also, four out of five major spliceosomal snRNAs (U1, U2, U4, U5) were down-regulated in the F1-γ group, indicating transcriptional and posttranscriptional alterations. Accordingly, the expression of the long intergenic noncoding RNAs *malat-1*—also known as *NEAT2* and well known for its interaction with several serine/arginine proteins, in order to drive the distribution and function of splicing factors in the nuclear speckles—was altered in the F1 offspring. Finally, DE piRNA clusters were associated to nine transposable elements (TEs), and they appeared to be expressed from the complementary strand of the associated TEs, suggesting that their expression could be in response to the activation of TEs.

## 3. Radiation Modifiers in Zebrafish

For all the reasons explained so far, the zebrafish embryo is a suitable model for large-scale screening of therapeutic agents, such as IR and their modifiers, and it may be used for easy and rapid testing of novel radiation protectors and sensitizers ultimately intended for therapeutic use. An updated overview of studies examining these aspects is reported in Table 1 and discussed in the following section.

**Figure 1:**
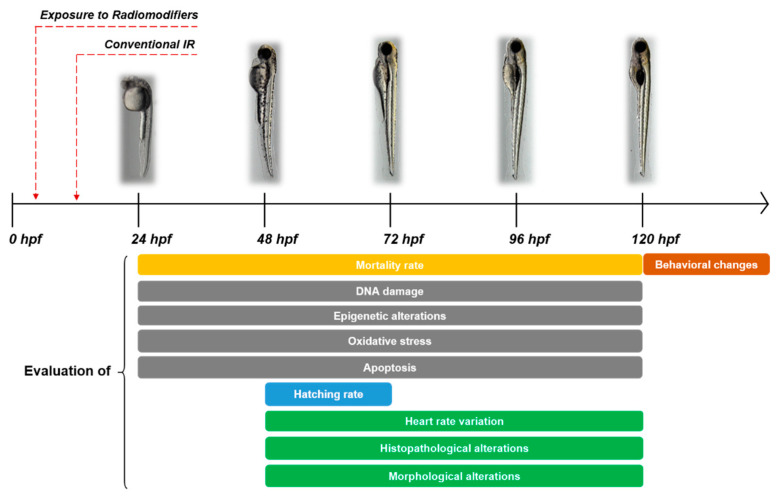
Diagrammatic representation of the experimental assays employed in the studies described in this review.

Figure 1 depicts the experimental assay generally employed in studies described below, involving the administration of radiomodifiers pre- [26,34,35,36,37,38,39,40,41,43,44,45,46,47,48,49,50] or post-IR treatment [42]. Their possible radiosensitizing or radioprotective properties are evaluated performing a series of analyses at key times during embryogenesis. In particular, the assessment of mortality, confirmed by the absence of blood circulation or spontaneous movements within the chorion, begins at 24 hpf. The search for characteristic morphological alterations—such as micropthalmia, spinal curvature, pericardial edema and the inhibition of yolk sac resorption—as well as the histopathological assessment, starts at 48 hpf, when there is completion of rapid morphogenesis of primary organ systems, cartilage development in head and pectoral fin [52]. Despite heart contraction beginning approximately at 24 hpf, heart rate evaluation also starts at 48 hpf, when a regular heartbeat is observed [53]. Although embryo hatching occurs asynchronously from 48 to 72 hpf, early hatching fish larvae are not more developmentally advanced than those remaining in their chorion. Consequently, the time of hatching is not always useful as an index of delayed staging due to a given treatment [52]. Whereas, during the hatching period, the embryo is usually at rest; the early larvae gradually begin to swim actively and produce swift escape responses, heralding the seeking of prey and feeding [52]. For this reason, evaluation of behavioral changes starts from 120 hpf, either by using a manual test, such as the touch-evoked response and locomotion assay [54], or by an automated video-tracking solution. Apoptosis assessment could be done directly through the immunofluorescence assay for CASP-3, Tunel assay or by in vivo staining with the fluorescent dye acridine orange [55,56]. To evaluate the extent of DNA damage, some researchers propose the search for ɣ-H2AX or 53BP1 foci by immunofluorescence assay, while others prefer to evaluate the variation in the expression level of key molecules belonging to specific DNA-repair pathways by means of immunofluorescence and Western blotting assay [57,58]. The same experimental approach is used to indirectly evaluate oxidative stress, by analyzing the expression levels of the main enzymes involved in the detoxification pathway or hydroxyl radical and superoxide anion scavenging activity [59].

This overview below is subdivided into distinct subparagraphs according to the main biological processes affected by a given radio-protector/sensitizer used, namely oxidative stress, DNA damage and apoptosis.

### 3.1. Oxidative Stress

ROS and reactive nitrogen species (RNS) are produced by intracellular water radiolysis, due to beam interaction. Free radicals, such as OH^•^, O_2_^•−^ and HO_2_^•^, play a central role in both DNA-damaging and -repair impairment, protein oxidation and lipid peroxidation, causing cytotoxicity associated with cell cycle arrest, apoptosis or necrosis [60]. On the other hand, ROS formation also induces adverse effects on normal cells and tissues, by means of inflammation, which is the main pathogenic consequence of RT *sequelae* [61]. Although the ROS-induced damage associated with irradiation depends on several factors, such as the LET value, the dose and the dose rate used during irradiation and the intrinsic radiosensitivity of target tissue [62], the accumulation of ROS is physiologically limited by specific cellular systems involving glutathione metabolism and dedicated enzymes, such as superoxide dismutase (SOD), catalase (CAT), and glutathione peroxidases [63].

Considering that this phenomenon can differentially influence the response to an RT treatment, some studies have the effects of in vivo pretreatment with radioprotective/radiosensitizing molecules involved in this process. For example, a 30 min pre-irradiation treatment of 24 hpf zebrafish embryos with the antihypertensive Todralazine significantly reduced the rate of morphological alterations due to IR, and protected embryos from lethality [34]. Indeed, while irradiation alone caused a 1.85-fold increase of ROS levels, Todralazine pretreatment reduced this value to 1.56-fold [34].

Similarly, it was observed that 3 h of pre-IR treatment with the fullerene derivative DF1 increased the survival rate of 6 dpf irradiated zebrafish juveniles from 40% to 85% [35]. Moreover, the incidence of the “cup” phenotype was reduced from 65% to 10% and from 70% to 35%, for exposure to 20 and 40 Gy, respectively. Additional evaluations highlighted a protective action on renal and nervous system, with a clearance increase of 30% and a reduction of developing nerve cells in zebrafish. ROS levels, evaluated at zero and 2 h post-IR, decreased in the pre-treated embryos, with values of 0.7 vs. 1 for the former time point and 1.2 vs. 2.3 for the other [35].

A variation in the hydroxyl radical and superoxide anion-scavenging activity was also evaluated by Dimri L. et al. [36], who studied the effect induced in 20 hpf embryos under 30 min of pre-IR treatment with the anesthetic prilocaine hydrochloride, an intermediate-acting local anesthetic of the amide type chemically related to lidocaine. First, the addition of this compound led to a more than 85% reduction in all mild adverse effects due to irradiation, and 60% in embryo mortality at 4 days post-irradiation (dpi). Moreover, prilocaine-treated embryos did show significantly enhanced erythropoiesis, suggesting a specific effect on the hematopoietic stem-cell lineage [36].

Lee WW et al. [37] examined the antioxidant effect of the aqueous extract from octopus ocellatus meat (OMA) on 24 hpf zebrafish embryos before irradiation. They found that the survival rate was markedly improved (75%–80%) in the pretreated and irradiated embryos, compared with the irradiated ones (40%), and that no abnormal tail bending was exhibited by pretreated and irradiated embryos. In addition, OMA pretreatment significantly reduced both ROS and NO levels in a dose-dependent manner [37], thus demonstrating that a simple and easily available food extract can protect against adverse effects induced by radiation.

In a distinct study, the protective activity of L-alpha glycerylphosphorylcholine (GPC) was tested in zebrafish embryos at 6 and 24 hpf [38]. In particular, while embryos irradiated with 20 Gy started to die at day 3 and had all died by day 5 post-irradiation, embryos pretreated with GPC and exposed to the same radiation dose were still alive at the same point in time. Histopathological assessment on irradiated fish highlighted severe alterations in the gastrointestinal system, including large amounts of mucus and catarrh in the intestinal flux, with goblet cells found in the intestinal mucous membrane, where cells had irregular shapes and larger hyperchromatic nuclei in a wider cytoplasm. All these alterations were attenuated and partially restored by GPC pretreatment, confirming the results of previous in vitro and in vivo studies about the GPC protective role against lethality and multi-organ morphological and histological impairment [64,65,66].

Another study reported that, in comparison with the irradiation-alone group, pre-exposure of 4 hpf zebrafish embryos to the antidiabetic phenformin hydrochloride significantly increased both their survival and hatching rates, and reduced the malformation incidence at 24 and 48 hpf, by preventing the deficit in spontaneous movement and heart rate [39]. In addition, phenformin pretreatment reversed the gene-expression variations associated with IR, restoring the normal cellular abundance of SOD, CAT, acetylcholinesterase and malondialdehyde, while leading to upregulation of BDNF and bcl-2 expression, and downregulation of p53, BAX and ɣ-H2AX [39].

Encouraging results were also reached with CO-releasing molecules (CORMs), widely known to alleviate toxicity from oxidative stress. For example, pretreatment of 4 hpf zebrafish embryos with CORM-3 increased their hatching rate and reduced the incidence of mortality at 72 and 120 hpf, compared with the only irradiated group [40]. The evaluation of spontaneous movement and larval behavior at 24 and 144 hpf highlighted that CORM-3 pretreatment afforded a significant behavioral advantage, compared with larvae from the only irradiated group. ROS levels were reduced by CORM-3 pretreatment, while SOD and CAT activities were increased. Acridine orange staining also revealed that apoptosis, induced by irradiation, was strongly reduced by CORM-3 pretreatment, likely due to the increase in bcl-2 and decrease in BAX, caspase-9 and caspase-3 mRNA levels. Being an efficient ROS scavenger, CORM-3 could suppress ROS production, alleviating the toxicity rate induced by X ray irradiation [40].

Further studies have reported preliminary findings. For example, Geng L. et al. evaluated the radiosensitizing effects of pretreatment with VJ115, an inhibitor of ENOX1’s NADH oxidase activity previously tested on Lewin’s lung carcinoma and HT29 xenograft models. Whereas IR exposure of 24 hpf zebrafish embryos did not affect their ability to hatch between 48 and 72 hpf, VJ115 pretreatment resulted in a dose-dependent hatching inhibition [41,66].

Similarly, exposure of 6 hpf-zebrafish irradiated embryos to the synthetic organic compound menadione (2-methyl-1,4-naphthoquinone) exacerbated the RT effect, as evidenced by higher developmental defects and higher TUNEL-staining in embryos exposed to the combined treatment [42]. However, additional study isrequired to confirm the potential therapeutic use of these agents as radiosensitizers.

### 3.2. DNA Damage

Being the most sensitive macromolecule to IR, DNA is certainly the main target of cellular damage. In general, minor DNA damage is thought to halt cell cycles allowing effective repair, while more severe damage can induce an apoptotic cell-death program [67].

Lally BE et al. used zebrafish embryos as a U251 xenograft model to test the in vivo radiosensitizing effect of the DNA repair inhibitor 4′-bromo-3′-nitropropiophenone (NS-123) [43]. In particular, 24 hpf embryos bearing fluorescently labelled U251 xenograft tumors were incubated with NS-123 for 14 h, before exposure to 10 Gy ɣ rays. As reported, NS-123 pretreatment specifically provoked a marked regression of the xenograft size, measured as extinction of the xenograft fluorescence at 3 or 5 dpf. Immunoblotting assays also revealed substantially higher levels of γ-H2AX, P-CHK2, P-DNA-PKcs and P-ATM in U251 cells that endured the combined treatment compared to irradiated experimental groups, suggesting that unrepaired DSBs accumulate in the presence of NS-123. Accordingly, control DMSO-treated U251 cells showed almost complete repair of double strand breaks (DSBs) at 24 h (∼9% of their initial damage), while NS-123-treated cells showed ∼25% of their initial damage [43].

Temozolomide is a drug widely known for its antineoplastic cytotoxicity related to the perturbation of DNA repair [68]. Using an approach similar to that described for NS-123, it has been reported that the treatment of zebrafish embryos bearing fluorescently labelled glioma cell transplants with temozolomide, followed by exposition to 10 Gy γ-rays, significantly decreased the tumor size at 3 and 5 dpf, with complete eradication of tumor masses in 21% of the embryos [26].

The most dangerous lesions directly inflicted by IR on DNA are DSBs, which can increase the recurrence of chromosomal translocation and genome mutation [69]. DSBs are repaired by two main mechanisms, namely homologous recombination and nonhomologous end joining (NHEJ), both involving the concurrent triggering of complex signaling networks often connected to each other [58,70]. Notably, the spatial expression pattern of zebrafish Ku70, a component of the NHEJ pathway, suggests a role for this DNA repair protein in the differentiation of neural tissue [17,71]. In order to evaluate its potential sensitizing role, Ku70 knockdown was imposed by microinjection of antisense morpholino oligonucleotides (MO) into the yolk of one-cell zebrafish embryos [44]. In particular, two distinct oligonucleotides were designed, one complementary to the Ku70 translation initiation site (atgMO) and the other complementary to the splice donor sequence of intron 2 (sd2MO), and their effects were compared to those of a control oligonucleotide. Injected embryos were irradiated (50 cGy ɣ rays) at 6 hpf and TUNEL-assayed at 24 hpf. In contrast wth non-irradiated control embryos, irradiation of embryos microinjected with atgMO resulted in profound developmental defects and cell death, associated with elevated amounts of TUNEL-positive cells in the central nervous system, retina and tail. Similar effects were also detected in embryos injected with sd2MO, although their phenotype was consistently less severe, and the overall amount of TUNEL staining was reduced [44]. These results suggest that Ku70 knockdown is sufficient to modify radiosensitivity in vivo, and that Ku70 plays a crucial role in protecting the differentiating nervous system from radiation-induced DNA damage during zebrafish embryogenesis.

In this respect, another research group tested the apoptotic effect on neural tissue caused by exposure of 24 hpf zebrafish embryos to high doses of IR, in the range of 8–15 Gy [45]. In particular, they performed a recessive genetic screening, using 8 Gy X rays, and identified a number of mutations that could sensitize embryos to IR, among which the main character was the radiosensitizing mutation 7 (rs7). This mutation causes major deterioration of neural tissue, leading to a small-head and curled-tail phenotype by 2 dpf, and death by the end of 3 dpf. To verify the relation between neural cell death and apoptosis in irradiated rs7 mutants, the authors performed immunofluorescence at 6 h post-IR to detect active caspase-3. In this assay, irradiated rs7 mutants showed a dramatic increase in cell death in the neural tissue when compared to wild-type irradiated embryos, with a 95-fold increase in active-Caspase-3 staining. Having identified that the rs7 mutation consisted in the presence of premature stop codon in the coiled-coil domain containing gene 94 (ccdc94), they injected embryos, derived from a cross between rs7 heterozygotes with synthetic mRNA encoding either zebrafish ccdc94, human ccdc94 or egfp (as a control), and exposed the resulting embryos to 8 Gy X rays. Importantly, embryos injected with zebrafish or human ccdc94 mRNA fully rescued the rs7 radiosensitization phenotype [45]. The protein expressed by ccdc94 is a functional component of the Prp19 complex, that, in vertebrate cells, has established roles in pre-mRNA-splicing and DNA-repair pathway, regulating the p53 expression. Therefore, this study shows that depleting components of the Prp19 complex sensiti to DNA damage.

A more recent study reported that embryos derived from homozygous tp53M214K zebrafish mutants are associated with a complete lack of dorsal tail curvature and cell-death induction in response to IR [46]. Importantly, inhibitors of checkpoint kinase 1, such as oxfendazole, efficiently radiosensitized tp53M214K mutants with minimal toxicity, and their effect was shown to be apoptotic in nature, as revealed by acridine orange, TUNEL and active CASPASE-3 stains of 48 hpf embryos. In addition, it was associated with an increase in DNA damage in a dose- and time-dependent manner, with maximal efficacy when administered 0–4 hpi. Moreover, the similarity-ensemble approach—a target-prediction algorithm—identified, as novel targets, the interleukin-1 receptor-sssociated kinases (IRAK) 1 and 4, whose inhibition by IRAK1/4 inhibitor (IRAK1/4i) was able to radiosensitize tp53M214K embryos. IRAK1 is known to be an effector of IL-1R and TLRs in innate immune signaling, acting in pro-survival and inflammatory responses to pathogens. Indeed, experimental inhibition of one of its additional candidate targets, as the peptidyl-prolyl cis/trans isomerase PIN142, radiosensitized zebrafish tp53M214K mutants, with similar potency to that of IRAK1/4i [46].

### 3.3. Apoptosis

Although most of DNA damage is correctly repaired, it is well known that the repair process often results in small deletions or insertions at the breakpoints, loss of heterozygosity and chromosomal translocations [72,73,74]. These rearrangements might be inconsequential or they may promote cell death or cancer initiation, depending on their location in the genome [75].

In a recent study, E.Y. Kong et al. evaluated the influence of exogenous NO, generated using the NO donor S-nitroso-N-acetylpenicillamine (SNAP), on X-ray-induced targeted and non-targeted bystander effects in zebrafish embryos [47]. They found that SNAP treatment of 5 hpf zebrafish embryos, followed by irradiation with 75 mGy X rays, resulted in a slight reduction in the mean number of apoptotic events, detected by acridine orange vital staining at 24 hpf, compared with that obtained in the only irradiated embryos at the same stage. However, when unirradiated 3 hpf embryos were pretreated with SNAP, transferred into fresh culture medium, and eventually partnered with the only irradiated embryos until they reached the 24 hpf stage, the mean number of apoptotic events on the SNAP treated embryos was significantly lower [47].

Duffy KT et al. studied the p53 role, testing the effect of pre-IR treatment of early zebrafish embryos with hydroxylprolyl-phosphono peptide nucleic acid (HypNA-pPNA) hetero-oligomers targeting tp53 mRNA or with the inhibitor of p53 transactivation activity pifithrin-α (PFT-α) [48]. While 100% of only irradiated embryos showed a characteristic aberrant dorsal curvature at 48 hpf and a 100% mortality rate at 144 hpf, embryos pre-treated with HypNA-pPNA were normal and mostly viable at all IR doses (0–40 Gy) used. By contrast, embryos pretreated with PFT-α and irradiated, died by 120 hpf, highlighting different mechanisms of the two molecules on p53 related apoptosis.

In a distinct study, PFT-α was used in combination with PS-341, a radiosensitizer known to abrogate the TNFα-induced NF-κB activity [76,77]. A preliminary experiment showed that pre-IR exposure of 24 hpf zebrafish embryos with PS-341 consistently reduced the survival rate at 7 dpi [49]. Then, p53 was knocked down, by injecting antisense morpholino oligonucleotides in developing zebrafish embryos or exposing them to PFT-α, to inhibit the p53 transactivation activity before embryo irradiation. The latter treatment markedly improved zebrafish survival after irradiation, either alone or in combination with PS-341. RT-PCR analysis did not reveal increased mRNA levels of the p53 targets p21/WAF1, bax or mdm2 in PS-341 treated embryos, whereas IR led, as expected, to elevated transcript levels for these genes [49]. Thus, the molecular targets responsible for radiosensitization by PS-341 and their relationship to p53 remain to be identified.

Considering its ability to enhance radiation-induced apoptosis in in vitro assays, the mammalian pro-apoptotic BAD was overexpressed by Jette CA et al. as an mCherry-BAD fusion protein in zebrafish developing embryos, exposing them to 15 Gy ɣ rays at 24 hpf, and measuring CASP-3 activity [50]. As expected, irradiated embryos overexpressing mcherry-BAD showed a greater level of activated CASP-3 throughout the brain and spinal cord compared with only irradiated embryos. In addition, overexpression of a mCherry-BAD protein bearing a mutation in the BH3 domain led to apoptotic levels to those of only irradiated embryos, demonstrating that the BAD-dependent enhancement of radiation-induced apoptosis requires a functional BH3 domain.

McAleer MF et al. used zebrafish embryos to compare the effects on the radiation response of flavopiridol to those associated with cyclin D1 (ccnd1) knock down by antisense HypNApPNA oligomers [11]. Flavopiridol pretreatment resulted in a “curly up” phenotype at a lower radiation dose, compared with only irradiated embryos, and in dramatic increase in embryo lethality at 120 hpf. Similar outcomes were obtained following the injection of ccnd1-specific HypNA-pPNA oligomers, supporting the hypothesis that cyclin D1 inhibition is sufficient to account for the radiosensitizing effects of flavopiridol in zebrafish embryos.

## 4. Conclusions and Perspectives

Over the past decades, the combination of chemotherapy and radiotherapy has become the standard modality for treatment for many solid tumors at certain stages, from brain to pelvis, head and neck, bladder, anal and advanced cervical cancers, and, to varying degrees, in gastric and pancreatic cancer, glioblastoma and sarcomas, improving overall survival [78].

In general, chemotherapy and other radiosensitizers, administered as neoadjuvant treatment to RT, are used with the aim of obtaining a modification of the slope of the dose-response curve and to prevent or delay metastasis, thanks to their systemic effects. The drugs’ and radiations’ action mechanisms can be different, thus the two therapies could act independently. However, in most cases, their effects are additive (combined action on the same target, with radio-enhancing effect) or synergistic (where the final effect’s magnitude is more than can be explained additively).

In the combined chemo–radio-therapy treatment, the drug could have disparate molecular mechanisms; however, in various ways, they act by participating ino the (I) inhibition of the sublethal damage recovery; (II) inhibition of repopulation; (III) enhancing sensitivity of hypoxic cells; and (IV) promoting cell cycle synchronization in phases more sensitive to RI [79]

However, if the rationale for therapeutic combinations is to increase their local effect, the increase of cytotoxic effects is inevitable and, in some cases, not always tolerable, thus, in recent years the interest for more tolerable radiomodifiers has grown.

This review provides an updated overview of the state of the art on the use of the zebrafish embryo model for testing radiomodifier molecules in combination with RT. All examined studies confirm that the zebrafish is an excellent vertebrate model for experimental validation and optimization in the field of radiobiological research. Indeed, zebrafish have both a full complement of vertebrate-specific organs of radiobiological interest and an irradiation size useful for detailed investigations on RBE–LET correlation. Worth mentioning, zebrafish embryos are permeable to a wide range of molecules, and the use of fish-larvae xenografts, which allow us to assess tumorigenicity of human cancer cells irradiated in vivo [51].

A common finding from all the studies described in this review is that radioprotectors mainly act by reducing oxidative stress, while radiosensitizers act through many other and, often, related mechanisms. It follows that the identification and characterization of these mechanisms could be helpful for the development of novel combined therapeutic strategies aimed at minimizing the side effects induced by radiation on healthy tissue, while, concomitantly, improving the selective targeting of tumor tissues.

Remarkably, studies adequately exploring the transgenerational effects of IR in zebrafish are still missing. Future investigation on this point is needed, especially considering the growing use of radiation for cancer treatment in subjects of childbearing age, where the concept of genomic instability inherited from offspring must necessarily be explored. The finding of delayed and persistent hereditary effects caused by parental irradiation—mostly expressed as increased ROS formation, DNA damage and bystander effects in the offspring—highlights the need for further study to provide information on the potential transgenerational effects induced by RT protocols, as well as on the environmental impact of IR. Once again, zebrafish would be the best choice for in-depth study in this field, predominantly because of its conservation in DNA repair and epigenetic mechanisms.

## Figures and Tables

**Table 1:** Summary of the experimental conditions and effects observed using the radiomodifying agents described in this review. Each column, from left to right, highlights the following aspects: reagent name, reagent role (either radioprotector or radiosensitizer), concentration used, molecular pathway affected, radiation dose used, and references. * In this case, no concentration values are indicated because the reagent used was either a genetic mutation (radiosensitizing mutation 7) or a knocked-down gene (AEG-1).

Reagent	Role	Concentration	Affected Pathways	Radiation Dose	References
Flavopiridol + HypNA-pPNA	Radiosensitizer	0–500 nM + 5 mM	Cell cycle, Apoptosis	10–40 Gy ɣ rays	[11]
Temozolomide	Radiosensitizer	100 µM	DNA damage	10 Gy ɣ rays	[26]
Todralazine	Radioprotector	5 µM	Oxidative stress	20 Gy ɣ rays	[34]
Metoprolol	Radioprotector	5 µM	Oxidative stress	20 Gy ɣ rays	[34]
Fullerene derivative DF-1	Radioprotector	100 µM	Oxidative stress	20–40 Gy ɣ rays	[35]
Prilocaine hydrochloride	Radioprotector	10–40 µM	Oxidative stress	20 Gy ɣ rays	[36]
Octopus ocellatus meet (OMA)	Radioprotector	62.50–250 µg/mL	Oxidative stress	30 Gy ɣ rays	[37]
L-alpha-Glycerylphosphorylcholine (GPC)	Radioprotector	194–1944 µM	Oxidative stress, Fibrosis	5–20 Gy ɣ rays	[38]
Phenformin Hydrochloride	Radioprotector	25 µM	Oxidative stress, Apoptosis	4 Gy X rays	[39]
Ru(CO)3Cl-glycinate (CORM-3)	Radioprotector	10 µM	Oxidative stress, Apoptosis	4 Gy X rays	[40]
(Z)-(+/−)-2-(1-benzylindol-3-ylmethylene)-1-azabicyclo [2.2.2]octan-3-ol (VJ115)	Radiosensitizer	50 µM	Oxidative stress, Apoptosis	5–20 Gy ɣ rays	[41]
2-methyl-1,4-naphthoquinone (Menadione)	Radiosensitizer	0–10 µM	DNA damage	0.15–1.5 Gy ɣ rays	[42]
4′-bromo-3′-nitropropiophenone (NS-123)	Radiosensitizer	30 µM	DNA damage	10 Gy ɣ rays	[43]
Ku70 MOs	Radiosensitizer	4–5 ng	DNA damage	50 cGy ɣ rays	[44]
Radiosensitizing mutation 7 (rs7)	Radiosensitizer	- *	Apoptosis, DNA damage	8–15 Gy X rays	[45]
Oxofendazole	Radiosensitizer	20 µg/mL	Cell cycle, Apoptosis, DNA damage	15 Gy ɣ rays	[46]
GȌ6976	Radiosensitizer	20 µg/mL	Cell cycle, Apoptosis, DNA damage	15 Gy ɣ rays	[46]
S-nitroso-N-acetylpenicillamine (SNAP)	Radioprotector	20–100 µM	Apoptosis	75 mGy X rays	[47]
HypNA-pPNA 16-mer	Radioprotector	0.5 pmol	Cell cycle, Apoptosis	0–40 Gy X rays	[48]
pifthrin-a (PFTa)	Radioprotector	1 µM	Cell cycle, Apoptosis	0–40 Gy X rays	[48]
PS-341	Radiosensitizer	1 µM	Apoptosis	0–20 Gy X rays	[49]
mcherry-BAD mRNA	Radiosensitizer	100 ng/µL	Apoptosis	15 Gy ɣ rays	[50]
AEG-1 knockdown cells	Radiosensitizer	- *	Migration, invasion	0–10 Gy X rays	[51]

## Data Availability

Not applicable.

## Abbreviations

CAT	Catalase
Ccdc94	Coiled-coil domain containing gene 94
Ccnd1	Cyclin D1
CORM	CO-releasing molecule
DDR	DNA Damage Response
DMR	differentially methylated region
DNMT	DNA methyltransferases
dpi	days post irradiation
DSB	Double Strand Break
GPC	L-alpha glycerylphosphorylcholine
H3K27me3	Histone H3 trimethylation at lysine 27
H3K4me3	Histone H3 trimethylation at lysine 4
H3K9me3	Histone H3 trimethylation at lysine 9
hpf	hours post fertilization
HypNA-pPNA	Hydroxylprolyl-phosphono peptide nucleic acid
IRAK	Interleukin-1 Receptor-Associated Kinase
IR	Ionizing Radiation
LET	Linear Energy Transfer
MO	Morpholino Oligonucleotide
NHEJ	Non-Homologous End Joining
OMA	Octopus ocellatus Meat
PFT-α	Pifithrin-α
PTM	Post-translational modification
RBE	Relative Biological Effectiveness
RNS	Reactive Nitrogen Species
ROS	Reactive Oxygen Species
rs7	radiosensitizing mutation 7
RT	Radiation Therapy
SNAP	S-nitroso-N-acetylpenicillamine
SOD	Superoxide dismutase
